# Establishing the Oregon Center of Excellence for Behavioral Health and Aging: Results from the First Year

**DOI:** 10.1002/alz70860_107286

**Published:** 2025-12-23

**Authors:** Walter D Dawson, Allyson Stodola, Ellis Hews, Lindsey Smith, Paula Carder

**Affiliations:** ^1^ OHSU‐PSU School of Public Health, Portland, OR, USA; ^2^ Oregon Health & Science University, Portland, OR, USA; ^3^ Portland State University, Portland, OR, USA; ^4^ Oregon Alzheimer's Disease Research Center, Portland, OR, USA; ^5^ OHSU‐PSU School of Public Health, Portland, OR, Portland, OR, USA

## Abstract

**Background:**

Co‐occurring behavioral health conditions such as substance use disorders (SUDs) and serious mental illness (SMI) with Alzheimer's disease and other related dementias (ADRD) is an underrecognized public health issue. The prevalence of co‐occurring ADRD and behavioral health conditions is high and behavioral health conditions are a known risk‐factor for ADRD. The Oregon Older Adult Behavioral Health Initiative (OABHI) is an innovative state‐level program funded by the Oregon Health Authority to address gaps in services for older adults and people with physical disabilities with behavioral health (BH) issues. A state‐wide network of behavioral health specialists provide complex care consultation to health and human service organizations. A recent evaluation reported that 52% of consumers served by the OABHI presented with neurological/cognitive issues, and 13% were diagnosed with ADRD.

**Method:**

The Oregon Center of Excellence for Behavioral Health & Aging (OCEBHA) is a newly established, multi‐disciplinary academic center that aims to expand the workforce trained to provide behavioral health services for older adults, and to promote research, health policy, and programs that improve access to and quality of behavioral health services in Oregon. Though OCEBHA addresses behavioral health conditions widely, this Center is unique in its focus on serious mental illness (SMI) and substance use. This Center is the first state‐level center with this specific focus.

**Result:**

In the first year of operations, OCEBHA has developed an older adult behavioral health workforce assessment tool, a community asset map, and supported workforce development through the establishment of a leadership academy and hosting an annual conference.

**Conclusion:**

Innovative state‐level partners such as OCEBHA and the OABHI can help meet the unique needs of people living with occurring ADRD and behavioral health needs.

